# Highly efficient base editing in human tripronuclear zygotes

**DOI:** 10.1007/s13238-017-0459-6

**Published:** 2017-09-05

**Authors:** Changyang Zhou, Meiling Zhang, Yu Wei, Yidi Sun, Yun Sun, Hong Pan, Ning Yao, Wanxia Zhong, Yixue Li, Weiping Li, Hui Yang, Zi-jiang Chen

**Affiliations:** 1 Institute of Neuroscience, State Key Laboratory of Neuroscience, Key Laboratory of Primate Neurobiology, CAS Center for Excellence in Brain Science and Intelligence Technology, Shanghai Institutes for Biological Sciences, Chinese Academy of Sciences, Shanghai, 200031 China; 2 College of Life Sciences, University of Chinese Academy of Sciences, Beijing, 100049 China; 3 Shanghai Key Laboratory for Assisted Reproduction and Reproductive Genetics, Center for Reproductive Medicine, Ren Ji Hospital, School of Medicine, Shanghai Jiao Tong University, Shanghai, 200127 China; 4 Center for Reproductive Medicine, Shandong Provincial Hospital Affiliated to Shandong University, National Research Center for Assisted Reproductive Technology and Reproductive Genetics, The Key laboratory for Reproductive Endocrinology of Ministry of Education, Jinan, 250021 China; 5 Key Lab of Computational Biology, CAS-MPG Partner Institute for Computational Biology, Shanghai Institutes for Biological Sciences, Chinese Academy of Sciences, Shanghai, 200031 China; 6 Institute of Biomedical Sciences, Fudan University, Medical College, Shanghai, 200032 China; 7 Shanghai Center for Bioinformation Technology, Shanghai Industrial Technology Institute, Shanghai, 201203 China; 8 State Key Laboratory for Conservation and Utilization of Subtropical Agro-Bioresource, Guangxi University, Nanning, 530004 China

**Dear Editor,**

There are hundreds of disease-causing single-gene mutations, mainly caused by single-nucleotide substitutions or point mutations rather than small insertions/deletions (indels), and often there are no cures for these diseases. By introducing the CRISPR/Cas9 system into mouse zygotes, disease-causing mutations could be corrected, leading to the production of healthy adult animals (Long et al., 2014; Wang et al., 2013; Wu et al., 2013; Yang et al., 2013). Several studies have demonstrated that CRISPR/Cas9-mediated gene editing could also introduce precise genetic modifications in early human embryos (Kang et al., 2016; Liang et al., 2015; Tang et al., 2017). However, indels rather than single-nucleotide substitutions are obtained frequently, because most DNA double-strand breaks (DSBs) produced by programmable nucleases are repaired by error-prone non-homologous end-joining (NHEJ) rather than homologous recombination (HR) using a template donor DNA. Recently, base editors, composed of cytidine deaminase, Cas9 nickase (nCas9), and uracil DNA glycosylase inhibitor (UGI), have recently been developed to substitute a C at a target site with T without generating DSBs in plant, yeast, mouse zygotes, and human cells, and shown to be >100-fold more efficient than HR at generating point mutations (Kim et al., 2017a; Komor et al., 2016; Liang et al., 2017; Ma et al., 2016; Nishida et al., 2016; Zong et al., 2017). Moreover, the genome-targeting scope has been increased by using staphylococcus aureus CRISPR/Cas9 (SaCas9) with modified protospacer adjacent motif (PAM) recognition (Kim et al., 2017b). Yet, the efficiency and specificity of base editors has not been demonstrated in human embryos. Here, we report that both base editor 3 (BE3) using nCas9 and SaKKH-BE3 using SaKKH-nCas9 can introduce single-nucleotide substitutions efficiently in human tripronuclear (3PN) zygotes.

We first used BE3 (rAPOBEC1-nCas9-UGI) to induce point mutations in human β-globin (*HBB*), which associated with human diseases β-thalassemia (Fig. 1A and 1B). We expected to introduce a premature stop codon in *HBB* by G-to-A conversions at the target site. We carried out base editing in human 3PN zygotes by microinjection of BE3 mRNA and sgRNAs. The injected 3PN zygotes were cultured into 4 to 8-cell embryos and used for targeted-deep-sequencing analysis. Targeted point mutations were observed in 8 out of 19 (42%) embryos at the target site in the *HBB* gene, with mutation frequencies that ranged from 6% to 52% (Figs. 1C, 1D, 1K, and S1A). Targeted deep sequencing showed that 7 out of 8 embryos for *HBB* base editing contained a nonsense mutation at the target site, generated by a single G-to-A conversion (Figs. 1D and S1A).

**Figure 1:**
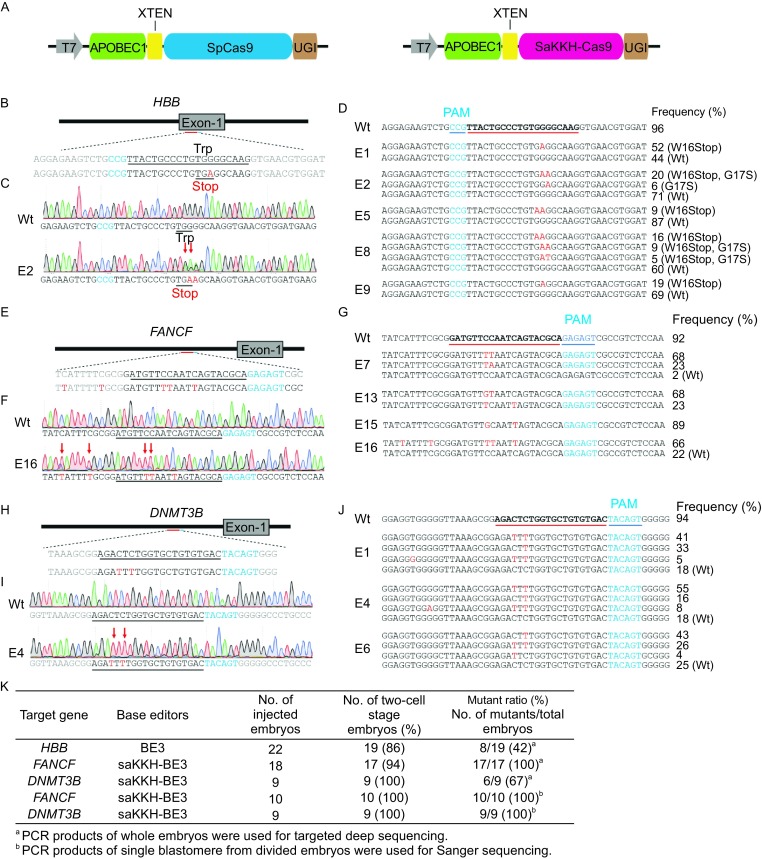
**Base editing in human 3PN zygotes using BE3 and SaKKH-BE3**. (A) Schematic diagram showing the structure of BE3 and SaKKH-BE3. XTEN, a linker; UGI, uracil glycosylase inhibitor. (B) The target sequence at the *HBB* locus. The sgRNA sequencing and the PAM sequence are shown in black and blue, respectively. The nucleotide substituted by BE3-mediated base editing is shown in red. (C) Sanger sequencing chromatograms of DNA from wild-type and *HBB*-E1 mutant embryo. The red arrow indicates the substituted nucleotide. The relevant codon identities at the target site are shown under the DNA sequence. (D) Alignments of mutant sequences from embryos with BE3-mediated editing at the *HBB* locus. The target sequence is underlined. The substitutions and PAM site are shown in red and blue, respectively. The column on the right indicates the percent of relevant genotype in total sequencing reads. Wt, wild-type. (E) The target sequence at the *FANCF* locus. The nucleotide substituted by SaKKH-BE3-mediated base editing is shown in red. (F) Sanger sequencing chromatograms of DNA from wild-type and *FANCF*-E16 mutant embryo. (G) Alignments of mutant sequences from embryos with SaKKH-BE3-mediated editing at the *FANCF* locus. (H) The target sequence at the *DNMT3B* locus. The nucleotide substituted by SaKKH-BE3-mediated base editing is shown in red. (I) Sanger sequencing chromatograms of DNA from wild-type and *DNMT3B*-E4 mutant embryo. (J) Alignments of mutant sequences from embryos with SaKKH-BE3-mediated editing at the *DNMT3B* locus. (K) Summary of base editing by BE3 and SaKKH-BE3 in human 3PN zygotes

To broaden the genome-targeting scope of base editors, we used the recently reported SaKKH-BE3 that relaxes the variant’s PAM requirement to NNNRRT (Fig. 1A). Targeted deep sequencing on the injected embryos revealed that 17 out of 17 (100%) or 6 out of 9 (67%) embryos carried targeted point mutations at the target site in the *FANCF* or *DNMT3B* gene, respectively (Fig. 1E–K). Note that we observed very low percentage (<5%) of wild-type (Wt) allele in 5 *FANCF* mutant embryos (*FANCF*-E2, E7, E9, E11, and E17) and no Wt allele in 3 *FANCF* mutant embryos (*FANCF-*E13, E14, and E15), indicating high base-editing efficiencies in human 3PN embryos using SaKKH-BE3 (Figs. 1G, S1B, and S1C). Targeted deep sequencing showed that a C-to-T conversion was the major mutagenic pattern at all three target sites, with frequencies range from 78.8% to 98.5% (Fig. S2). C-to-A or C-to-G conversions were also observed in 1 *HBB* (11%), 7 *FANCF* (70%), and 3 *DNMT3B* (50%) mutant embryos (Figs. 1 and S1). We also found C–T conversion on the upstream or downstream of the sgRNA target site in 0 *HBB* (0%), 10 *FANCF* (59%), and 3 *DNMT3B* (50%) mutant embryos (Fig. S2), consistent with previous studies (Kim et al., 2017a; Kim et al., 2017b; Komor et al., 2016). Using engineered base editors containing mutated cytidine deaminase domains, such as YE1-BE3, may narrow the width of the editing window (Kim et al., 2017b).

To avoid the PCR bias, we further examined 4- to 8-cell embryos with *FANCF* or *DNMT3B* base editing at the single-cell level. Single blastomeres of 4- to 8-cell embryos were isolated and picked up under the microscope for PCR amplification and Sanger sequencing. We found that 10 out of 10 (100%) or 9 out of 9 (100%) embryos carried targeted point mutations at the target site in the *FANCF* or *DNMT3B* gene, respectively (Figs. 1K, S3, and S4). Based on single-cell sequencing reads, 79% or 83% alleles carried targeted point mutations in the *FANCF* or *DNMT3B* (Fig. S5). Among these mutant embryos, two *FANCF* base-editing embryos (*FANCF*-E20, E24) and two *DNMT3B* base-editing embryos (*DNMT3B*-E11, E14) contained only targeted point mutations (Figs. S3 and S4). C-to-T conversion was the major mutagenic pattern, and C-to-A or C-to-G conversions were also observed in *FANCF* and *DNMT3B* mutant embryos (Fig. S5). Furthermore, compared with CRISPR/Cas9-mediated gene editing (Kang et al., 2016), although 7 out of 10 *FANCF* mutant embryos contained indels alleles (Fig. S3), the percentage of total DNA alleles with indels was very low (13% for *FANCF* and 0% for *DNMT3B*) (Fig. S5D). Further optimizing base editors with inactive Cas9 mutant or Cpf1 mutant may reduce the indels to a lower level.

Finally, to assess base editors off-target effects, we performed whole genome sequencing (WGS) to identify SaKKH-BE3 off-target mutations in the three *FANCF* mutant embryos (*FANCF*-E28, E29, and E30) (Fig. S6). Of 1,187 possible off-target sites that differ from the on-target site by up to 5 mismatches, we observed just 1 potential off-target site in 1 out of 3 *FANCF* mutant embryos (Fig. S6). Taken together, these results indicate that BE3 did not induce significant off-target alterations in gene-edited human embryos.

In summary, our results show that microinjection of BE3 or SaKKH-BE3 mRNA resulted in efficient and precise base editing in human 3PN zygotes. These results demonstrate that base editors can be used for correcting genetic defects in human embryos in the future.

## Electronic supplementary material

Below is the link to the electronic supplementary material.

Supplementary material 1 (PDF 1555 kb)
