# Supplementary material for: Highly efficient base editing in human tripronuclear zygotes
Source: Protein Cell. 2017 Sep 5;8(10):772–5. doi: 10.1007/s13238-017-0459-6 (PMC5636752; doi:10.1007/s13238-017-0459-6)
Supplement: Supplementary file 1 — Supplementary material 1 (PDF 1555 kb) [file 13238_2017_459_MOESM1_ESM.pdf]

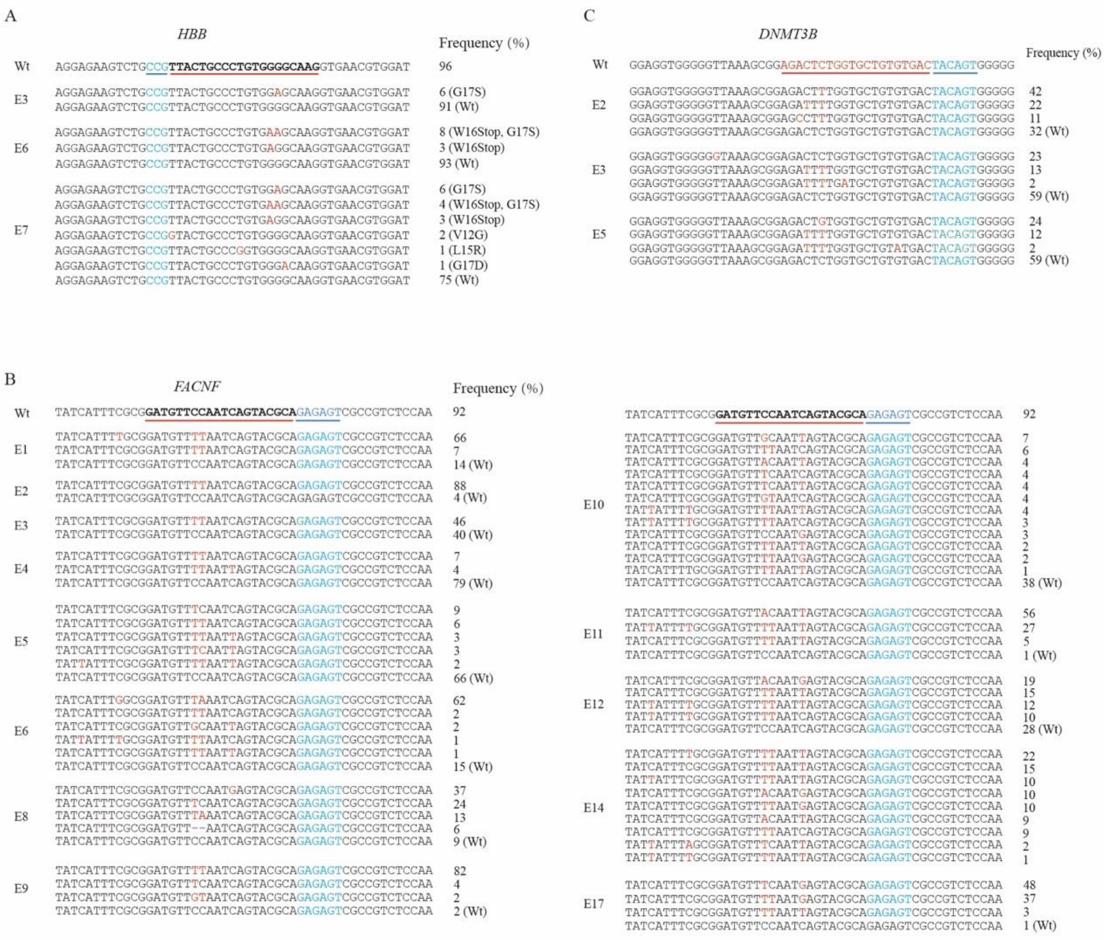

Figure S1. Targeted deep sequencing from embryos with BE3-mediated and SaKKH-BE3-mediated editing. (**A**) Alignments of mutant sequences from embryos with BE3-mediated editing at the *HBB* locus. (**B** and **C**) Alignments of mutant sequences from embryos with BE3-mediated editing at the *FANCF* (**B**) and *DNMT3B* (**C**) loci.

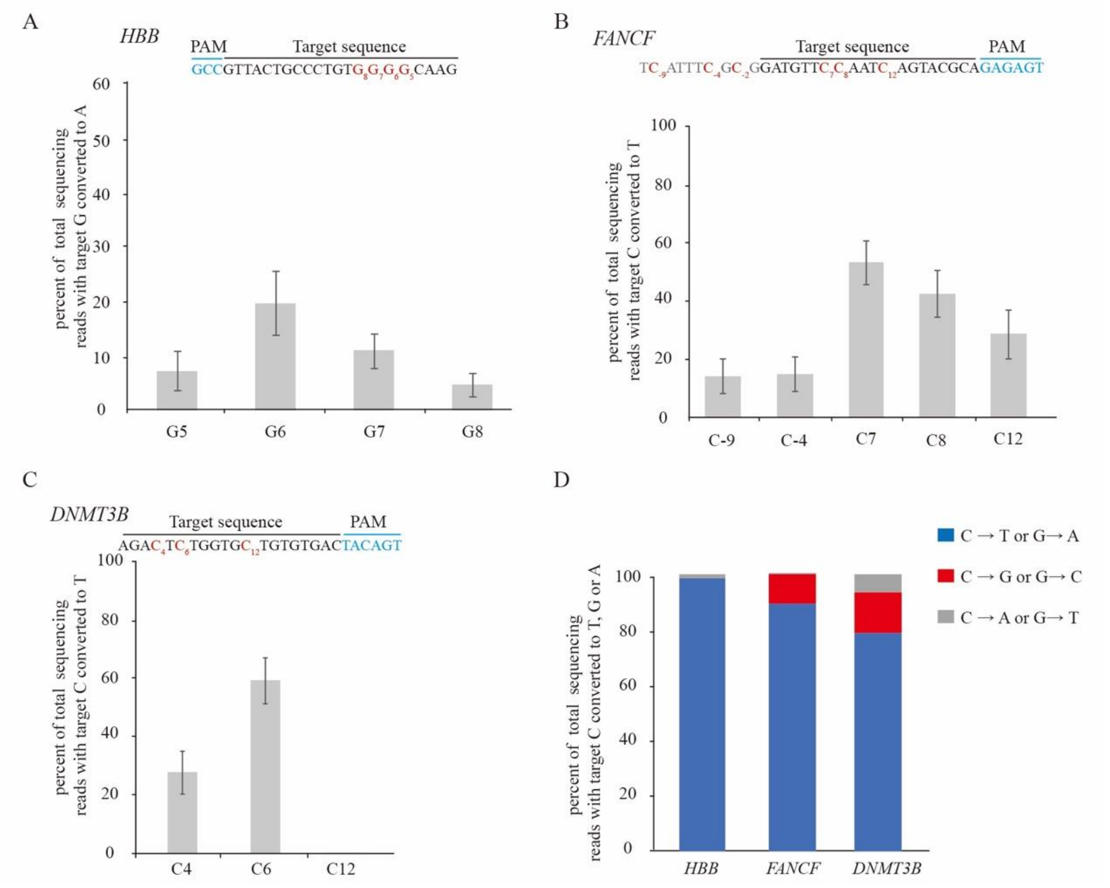

Figure S2. Targeted deep sequencing analysis on the base-editing embryos. (**A** to **C**) The percentage of total DNA sequencing reads with G converted to A at the target positions indicated on the *HBB* locus (**A**) or C converted to T at the target positions indicated on the *FANCF* (**B**) and *DNMT3B* (**C**) loci are shown. The results were presented as mean ± SD. (**D**) The percentage of total sequencing reads with target G converted to A, T or C, or C converted to T, G or A.

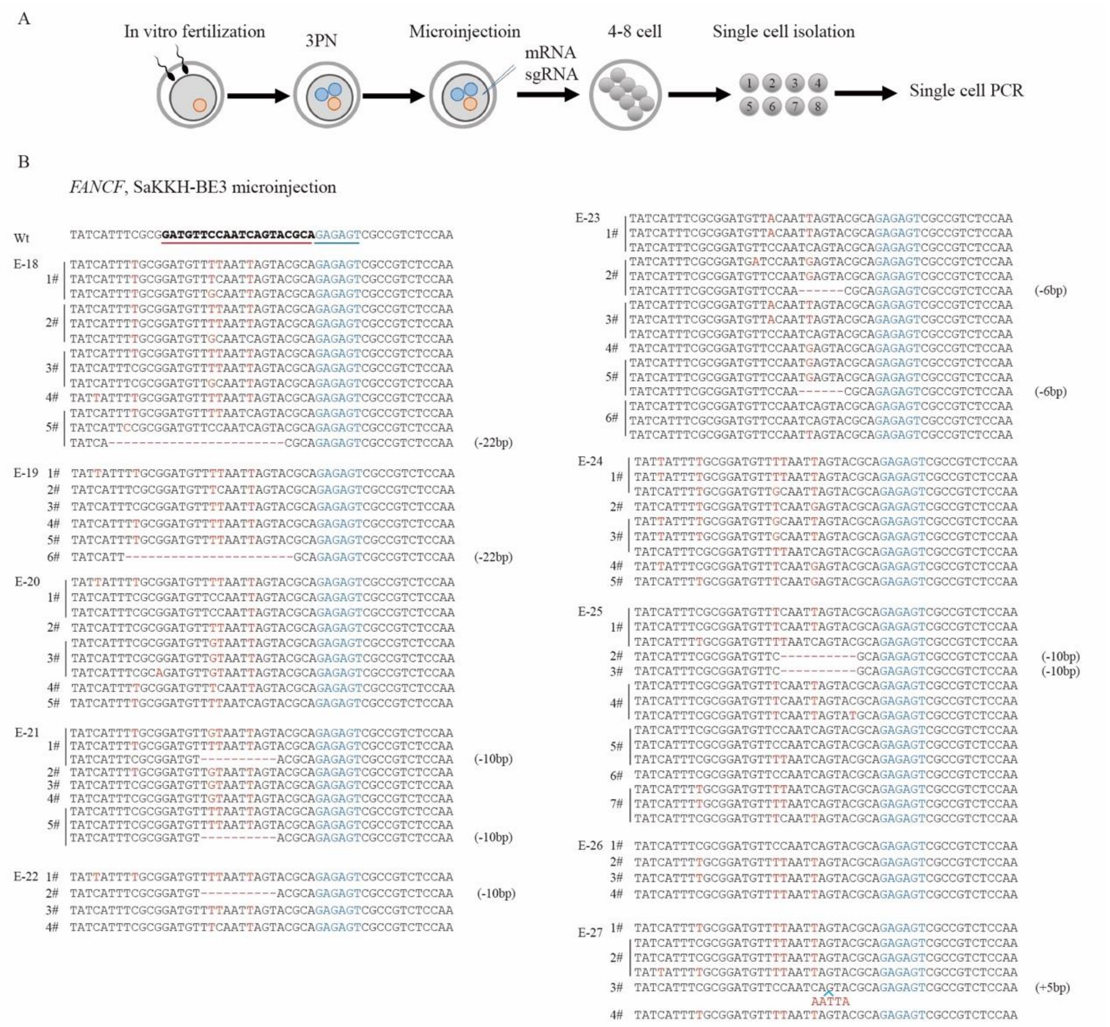

Figure S3. Single-cell analysis on embryos with SaKKH-BE3-mediated base editing at the *FANCF* locus. (**A**) Schematic diagram of experimental design. (**B**) Alignments of mutant sequences from embryos with SaKKH-BE3-mediated editing at the *FANCF* locus.

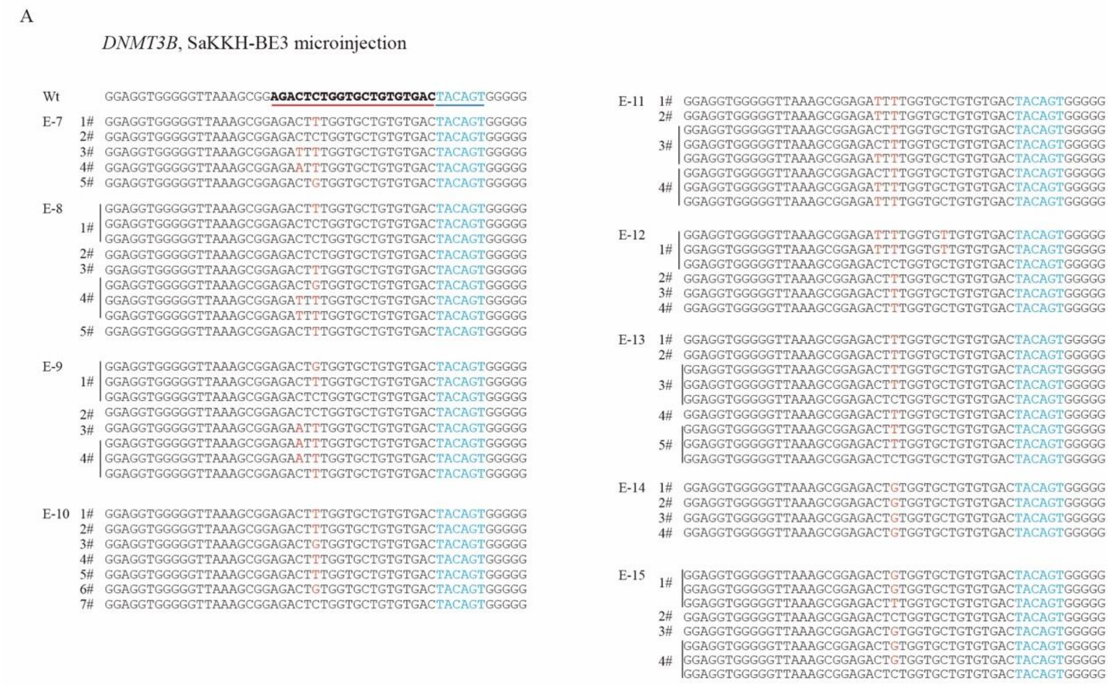

Figure S4. Single-cell analysis on embryos with SaKKH-BE3-mediated base editing at the *DNMT3B* locus. (**A**) Alignments of mutant sequences from embryos with SaKKH-BE3-mediated editing at the *DNMT3B* locus.

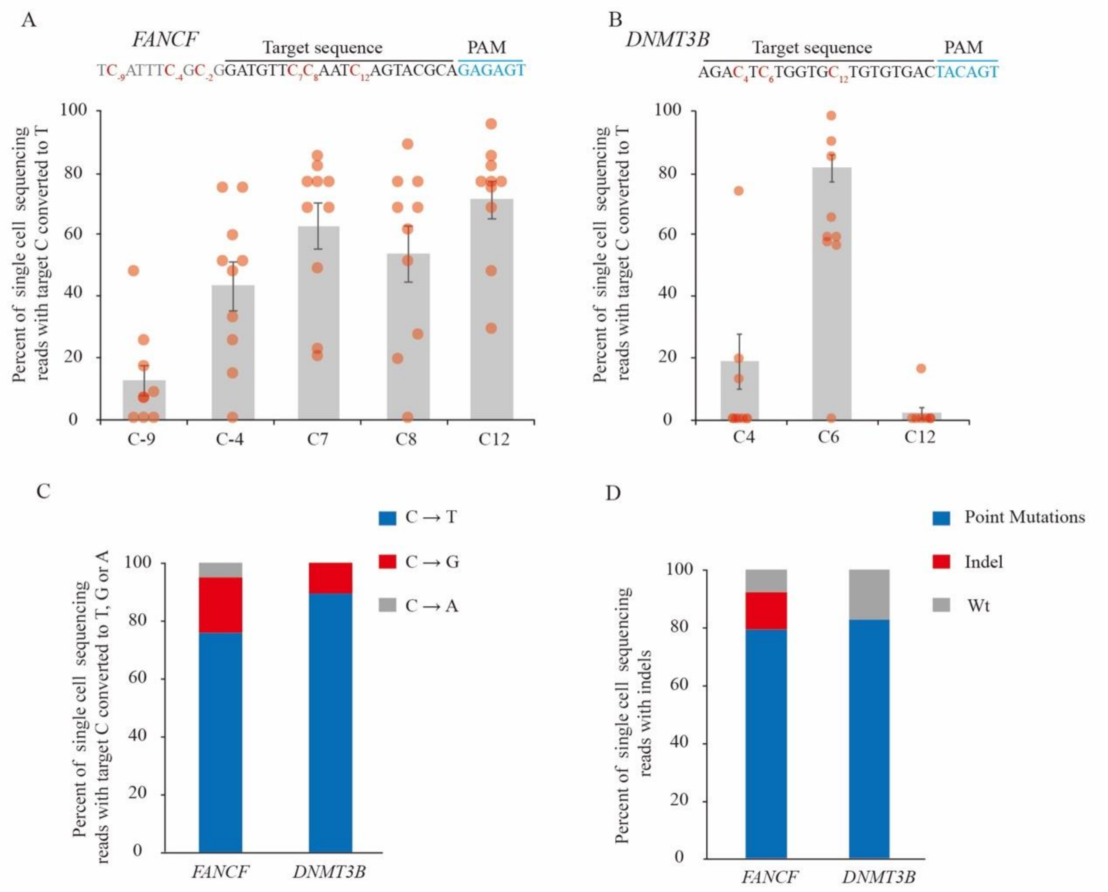

Figure S5. Single-cell analysis on embryos with SaKKH-BE3-mediated base editing. (**A** and **B**) The percentage of total DNA alleles with C converted to T at the target positions indicated on the *FANCF* (**A**) and *DNMT3B* (**B**) loci are shown. Each data point represents an individual embryo. The results were presented as mean ± SD. (**C**) The percentage of total DNA alleles with C converted to T, G or A. (**D**) The percentage of total DNA alleles with point mutations, indels or no mutations.

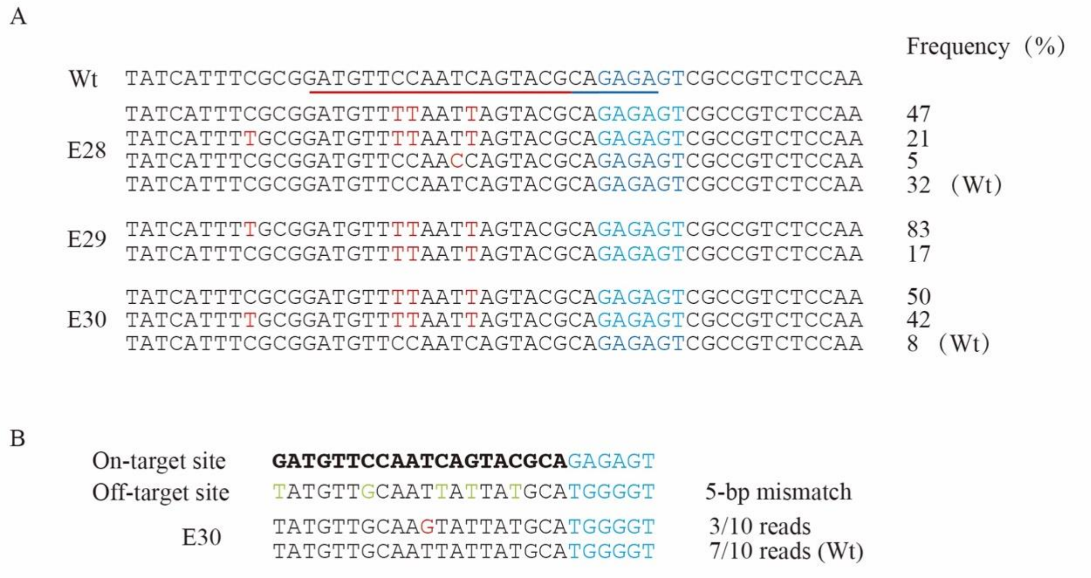

C

Summary of Indels and Single Nucleotide Variants (SNV) Detected by Whole Genome Sequencing

	E28	E29	E30
Total variants (SNPs + small indels)	3391315	3290273	3E+06
Raw SNVs	2911290	2814719	3E+06
Post filtering dbSNPs	161751	162035	142231
Reads number >2 SNV	52966	48758	47567
Post Low-complexity filter	25834	26062	26290
5-bp mismatch with NNNRRT PAM (1187)	1	0	2
On-target SNVs	1	1	1
Sample-specific SNVs	0	0	1
Raw indels	480025	475554	483673
Reads number >2 indel	342694	345114	365362
Post Low-complexity filter	94325	89562	96587
Post Homopolymeric filter	39645	35986	40644
5-bp mismatch with NNNRRT PAM (1187)	1	1	2
Sample-specific indels	0	0	0

Figure S6. Off-target analysis on embryos with SaKKH-BE3-mediated base editing at *FANCF* locus. (**A**) Point mutations at on-target site in three embryos with SaKKH-BE3-mediated base editing based on whole-genome-sequencing analysis. (**B**) A potential off-target site with 5-bp mismatch to the on-target site is shown. (**C**) Summary of indels and single nucleotide variants (SNV) detected by whole genome sequencing.

Table S1. The sgRNA sequences and primers

The sgRNA sequences.

Locus	Sequence(5'-3')
HBB	CCGTTACTGCCCTGTGGGGCAAG
FANCF	GATGTTCCAATCAGTACGCAGAGAGT
DNMT3B	AGACTCTGGTGCTGTGTGACTACAGT

Primers sequences used to generate the template for sgRNA *in vitro* transcription.

HBB	TAATACGACTCACTATAGGGCTTGCCCCACAGGGCAGTAAGTTTTAGAGCTAGAAATAG
FANCF	TAATACGACTCACTATAGGGGATGTTCCAATCAGTACGCAGTTTTAGTACTCTGGAAAC
DNMT3B	TAATACGACTCACTATAGGGAGACTCTGGTGCTGTGTGACGTTTTAGTACTCTGGAAAC
BE3 sgRNA-R	AAAAGCACCGACTCGGTGCC
SaKKH-BE3 sgRNA-R	AAAAATCTCGCCAACAAGTTG

Primer used for genotyping.

HBB	Outer F: ACATTTGCTTCTGACACAACTGT
	Outer R: GGTCCAAGGGTAGACCACCAG
	Inner F: GTTCACTAGCAACCTCAAACAG
	Inner R: CCAGCAGCCTAAGGGTGGGA
FANCF	Outer F: AGCGCGGAGACGTTCATGAC
	Outer R: GCTAATCCCGGAACTGGACCC
	Inner F: ACTGGCATCATCTCGCACGT
	Inner R: CCCTCTTGCCTCCACTGGTT
DNMT3B	Outer F: GGTTCAATGGTCATCCCAGGG
	Outer R: CCAGTGAAATCACCCTGGGG
	Inner F: TCATCCCAGGGCAGAGGTGG
	Inner R: CCTGGGGGATCAGAAGCCCT

Primers used for targeted deep sequencing

HBB	Primer-F-1	GACAGACAACTGTGTTCACTAGCAACC
	Primer-F-2	GACAGTCAACTGTGTTCACTAGCAACC
	Primer-F-3	GACAGCCAACTGTGTTCACTAGCAACC
	Primer-F-4	GACAGGCAACTGTGTTCACTAGCAACC
	Primer-F-5	GACAAACAACTGTGTTCACTAGCAACC
	Primer-F-6	GACAAT AACTGTGTTCACTAGCAACC
	Primer-F-7	GACAACCAACTGTGTTCACTAGCAACC
	Primer-F-8	GACAAGCAACTGTGTTCACTAGCAACC
	Primer-R	CCAGCAGCCTAAGGGTGGGA

FANCF	Primer-F-1	GAGAGACACGGATAAAGACGCTGGGAGA
	Primer-F-2	GAGAGTCACGGATAAAGACGCTGGGAGA
	Primer-F-3	GAGAGCCACGGATAAAGACGCTGGGAGA
	Primer-F-4	GAGAGGCACGGATAAAGACGCTGGGAGA
	Primer-F-5	GAGAAACACGGATAAAGACGCTGGGAGA
	Primer-F-6	GAGAATCACGGATAAAGACGCTGGGAGA
	Primer-F-7	GAGAACCACGGATAAAGACGCTGGGAGA
	Primer-F-8	GAGAAGCACGGATAAAGACGCTGGGAGA
	Primer-F-9	GAGATACACGGATAAAGACGCTGGGAGA
	Primer-F-10	GAGATTCACGGATAAAGACGCTGGGAGA
	Primer-F-11	GAGATCCACGGATAAAGACGCTGGGAGA
	Primer-F-12	GAGATGCACGGATAAAGACGCTGGGAGA
	Primer-F-13	GAGACACACGGATAAAGACGCTGGGAGA
	Primer-F-14	GAGACTCACGGATAAAGACGCTGGGAGA
	Primer-F-15	GAGACCCACGGATAAAGACGCTGGGAGA
	Primer-F-16	GAGACGCACGGATAAAGACGCTGGGAGA
	Primer-F-17	GAGTGACACGGATAAAGACGCTGGGAGA
	Primer-R	GGTGCTGACGTAGGTAGTGC
DNMT3B	Primer-F-1	GATAGAGCTCAGCCTGAGTGTTGAGG
	Primer-F-2	GATAGTGCTCAGCCTGAGTGTTGAGG
	Primer-F-3	GATAGCGCTCAGCCTGAGTGTTGAGG
	Primer-F-4	GATAGGGCTCAGCCTGAGTGTTGAGG
	Primer-F-5	GATAAAGCTCAGCCTGAGTGTTGAGG
	Primer-F-6	GATAATGCTCAGCCTGAGTGTTGAGG
	Primer-R	TGGTTGCCCACCCTAGTCATT

Materials and Methods

Source of tripronuclear human zygotes

Our research was approved by the Institutional Review Board of Reproductive Medicine of Shandong Provincial Hospital, and informed consent was obtained from all the participants.

Production of mRNA and sgRNA

The pCMV-BE3 and pCMV-SaKKH-BE3 were obtained from Addgene (Cat. No.73021 and 85170). The pCMV-BE3 and pCMV-SaKKH-BE3 plasmid were linearized with BbsI and used as the template for *in vitro* transcription (IVT) using the mMESSAGE mMACHINE T7 Ultra kit (Life Technologies). T7 promoter was added to sgRNA template by PCR amplification of px330, using primers listed in Table S1. The T7-sgRNA PCR product was purified and used as the template for *in vitro* transcription using MEGA shortscript T7 kit (Life Technologies). The BE3, SaKKH-BE3 and sgRNA were subsequently purified with the MEGAclear kit (Life Technologies) and eluted in RNase-free water.

Identification and collection of human tripronuclear (3PN) embryos

Mature oocytes were inseminated in fertilization medium (vitrolife, Sweden) 4h after retrieval by conventional in *vitro* fertilization (IVF). Fertilization status was checked 16~19h after insemination and normal fertilization was assessed by the presence of two clear pronuclei. Abnormal fertilized oocytes with three clear pronuclear were selected for further experiments.

Tripronuclear human zygote injection

The 3PN zygotes were transferred into HTF medium (Quinn's AdvantageTM Medium with HEPES). They were injected with BE3 or SaKKH-BE3 (100 ng/μl) and sgRNA (50 ng/μl) using the microinjector (FemtoJet, Eppendorf). The injected embryos were cultured in G1.5 media until day three. All embryos were maintained in a humidified incubator at 37°C, 5% CO^2, and 20% oxygen.

Genotyping and Targeted deep sequencing

Genome DNA was used for PCR amplification of target sites with primers listed in Table S1. PCR products were sequenced directly using primers from Table S1 to confirm the presence of double peaks and mutant embryos, and the mutant embryos were subjected to targeted deep sequencing. Target sites were amplified from genome DNA using Phanta Max Super-Fidelity DNA Polymerase (Vazyme Biotech Co., Ltd). The paired-end sequencing of PCR amplicons was performed by CloudHealth Genomics Co., Ltd using an HiSeq X Ten. A list of primers used for targeted deep sequencing is show in Table S1.

Single-cell PCR analysis

For picking up and transferring single cells, we used a glass capillary under a dissection microscope. 8~16 cell stage embryos were digested with acid Tyrode solution to remove the zona pellucida. Then transfer the embryos into 0.25% trypsin and gently pipette to separate the individual blastomeres. Finally, the blastomeres were washed in 0.25% trypsin for 8 to 10 times and transferred into a PCR tube. 1.5 μl lysis buffer containing 0.1% tween 20, 0.1% Triton X-100 and 4 μg/ml proteinase K was then pipetted at a temperature of 56 °C for 30min, followed by 95 °C for 5min. The products of the lysis program were used as templates in a nest PCR analysis.

Whole Genome Sequencing and off-target analysis

Whole genome amplification of the embryos was performed using the PEPLI-g midi kit (Qiagen). Briefly, embryos were digested with acid Tyrode solution to remove the zona pellucide and transferred into PCR tubes containing reconstituted buffer D2 (7 μl), and then incubated at 65 °C for 10min, before the addition of Stop solution (3.5 μl) and MDA master mix (40 μl) and incubation at 30°C for 8h. The DNA preparation was diluted with ddH_2O (1:30), and 1 μl of the diluted DNA was used for PCR analysis. Three samples (*FANCF*-E28, E29 and E30) were amplified and used for whole genome sequencing. Whole genome sequencing was carried out using Illumina HiSeq X Ten at mean coverages of 10X. Qualified reads were mapped to the human reference genome (hg19) by speedseq[1] (https://github.com/hall-lab/speedseq) with default parameters. All of the mapped data is available from the SRA under accession. FreeBayes[2] (v0.9.10) and LUMPY[3] (https://github.com/arq5x/lumpy-sv) were run on the aligned sequence files (BAM files) for single nucleotide variation (SNV), short indel detection and structural variation discovery. We firstly filtered SNVs which were included in dbSNP (v138). Both SNVs and indels were then filtered to remove variations with no more than two reads support. The SNVs and indels which located in low complexity regions including UCSC repeat regions and microsatellite sequences were removed. For the short indel variations, homopolymer with unit length greater than 7bp was also removed.

Potential off-target sites of sgRNAs were predicted as previously reported[4] (http://www.rgenome.net/cas-offinder/). We extracted all the off-target sites with no more than 5 mismatches for each sgRNA. We searched the single nucleotide variation (SNV) and short indel variations within the 26 bp predicted off-target sites, and variations occurred in more than one sample were also removed for further analysis. Variations after each filtering step were listed in the Supplementary Figure S6C.

Statistical Analysis

All statistical values were presented as mean ± SEM. Differences between datasets were judged to be significant at $p < 0.05$.

Reference

1. C Chiang, R M Layer, G G Faust, M R Lindberg, D B Rose, E P Garrison, G T Marth, A R Quinlan, and I M Hall. SpeedSeq: ultra-fast personal genome analysis and interpretation. *Nat Meth* (2015). doi:10.1038/nmeth.3505.
2. Garrison E, Marth G. Haplotype-based variant detection from short-read sequencing. *arXiv preprint arXiv:1207.3907 [q-bio.GN]* 2012.
3. Ryan M Layer, Colby Chiang, Aaron R Quinlan, and Ira M Hall. 2014. "LUMPY: a Probabilistic Framework for Structural Variant Discovery." *Genome Biology* 15 (6): R84. doi:10.1186/gb-2014-15-6-r84.
4. Bae S., Park J., & Kim J.-S. Cas-OFFinder: A fast and versatile algorithm that searches for potential off-target sites of Cas9 RNA-guided endonucleases. *Bioinformatics* **30**, 1473-1475 (2014).
